# Ceramides promote apoptosis for virus-infected lymphoma cells through induction of ceramide synthases and viral lytic gene expression

**DOI:** 10.18632/oncotarget.4759

**Published:** 2015-07-03

**Authors:** Lu Dai, Jimena Trillo-Tinoco, Aiping Bai, Yihan Chen, Jacek Bielawski, Luis Del Valle, Charles D. Smith, Augusto C. Ochoa, Zhiqiang Qin, Chris Parsons

**Affiliations:** ^1^ Research Center for Translational Medicine and Key Laboratory of Arrhythmias, East Hospital, Tongji University School of Medicine, Shanghai, China; ^2^ Department of Microbiology/Immunology/Parasitology, Louisiana State University Health Sciences Center, Louisiana Cancer Research Center, New Orleans, LA, USA; ^3^ Department of Medicine, Louisiana State University Health Sciences Center, Louisiana Cancer Research Center, New Orleans, LA, USA; ^4^ Department of Pathology, Louisiana State University Health Sciences Center, Louisiana Cancer Research Center, New Orleans, LA, USA; ^5^ Department of Biochemistry and Molecular Biology, Hollings Cancer Center, Medical University of South Carolina, Charleston, SC, USA; ^6^ Department of Drug Discovery/Biomedical Sciences, Hollings Cancer Center, Medical University of South Carolina, Charleston, SC, USA; ^7^ Department of Pediatrics, Louisiana State University Health Sciences Center, Louisiana Cancer Research Center, New Orleans, LA, USA

**Keywords:** KSHV, primary effusion lymphoma, sphingosine kinase, ceramide

## Abstract

Kaposi's sarcoma-associated herpesvirus (KSHV) is the etiologic agent for several human cancers including primary effusion lymphoma (PEL), a rapidly progressive malignancy arising preferentially in immunocompromised patients. With conventional chemotherapy, PEL continues to portend high mortality, dictating the development of novel therapeutic strategies. Sphingosine kinase 2 (SphK2) represents a key gatekeeper for sphingolipid metabolism, responsible for conversion of ceramides to sphingosine-1-phosphate (S1P). We have previously demonstrated that targeting SphK2 using a novel selective inhibitor, ABC294640, leads to intracellular accumulation of ceramides and induces apoptosis for KSHV-infected PEL cells, while suppressing tumor progression *in vivo*. In the current study, we sought to determine whether specific ceramide/dh-ceramide species and related ceramide synthases (CerS) impact viability for KSHV-infected PEL cells during targeting of SphK2. We found that several specific ceramide and dihydro(dh)-ceramide species and their associated CerS reduce PEL survival and tumor expansion *in vitro* and *in vivo*. Moreover, we found that dhC16-Cer induces PEL apoptosis in part through activation of KSHV lytic gene expression. These data further implicate bioactive sphingolipids in regulation of PEL survival, and provide justification for future studies evaluating clinically relevant ceramide analogs or mimetics for their potential as therapeutic agents for PEL.

## INTRODUCTION

Sphingolipids are a family of membrane lipids regulating the fluidity and subdomain structure of lipid bilayers [1, 2]. Ceramides are composed of a sphingosine base and amide-linked acyl chains of varied length [3]. Endogenous ceramide can be generated via *de novo* synthesis by ceramide synthases (CerS) [4, 5], or through the metabolism of other complex sphingolipids regulated by specialized enzymes [1, 2, 6]. Ceramides are hydrolyzed to generate sphingosine which is subsequently phosphorylated by one of two sphingosine kinase isoforms (SphK1 or SphK2) to generate sphingosine-1-phosphate (S1P) [1, 7-9]. Bioactive sphingolipids, including ceramides and S1P, act as signaling molecules to regulate apoptosis and tumor cell survival [1]. In contrast to the anti-apoptotic function of S1P, most endogenous long-chain ceramides are thought to induce cell death preferentially [7]. Over the past two decades, targeting bioactive sphingolipids has evolved as a promising therapeutic approach for cancer treatment [10].

A significant proportion of human cancers are attributable to viruses, including the Kaposi's sarcoma-associated herpesvirus (KSHV) [11]. KSHV is a common etiologic agent for cancers arising preferentially in the setting of HIV infection or organ transplantation, including primary effusion lymphoma (PEL) and Kaposi's sarcoma (KS) [12-15]. PEL tumors are comprised of transformed B-cells harboring KSHV and exhibit a rapidly progressive course, with a median survival of approximately 6 months with standard chemotherapy [12, 16]. The role of sphingolipids in virus-associated malignancies remains largely unknown, although one recent study indicates that KSHV induces fatty acid synthesis to promote survival of endothelial cells [17]. Another recent study demonstrates that KSHV-microRNAs can induce metabolic transformation of latently infected endothelial cells, including decreasing oxygen consumption, increasing lactate secretion and glucose uptake, stabilizing HIF1α and decreasing mitochondria copy number [18]. We recently reported that targeting SphK2 using either RNA interference or a selective small-molecule inhibitor, ABC294640, induces caspase-mediated apoptosis for KSHV-infected PEL cells and suppresses PEL tumor progression *in vivo* [19]. We also found that targeting SphK2 increases the collective accumulation of ceramides (including bioactive dihydro (dh)-ceramides) while reducing S1P concentrations within KSHV-infected cells [19, 20]. However, specific mechanisms for virus-infected lymphoma cell death associated with disruption of sphingolipids biosynthesis have not been previously addressed. Therefore, we sought to determine whether individual ceramide species induced apoptosis during perturbations in lipid metabolism in PEL cells, and if so, whether this effect was associated with alterations in KSHV gene expression.

## RESULTS

### Targeting SphK2 results in accumulation of ceramide species and upregulation of corresponding ceramide synthases within PEL cells

**Figure 1:**
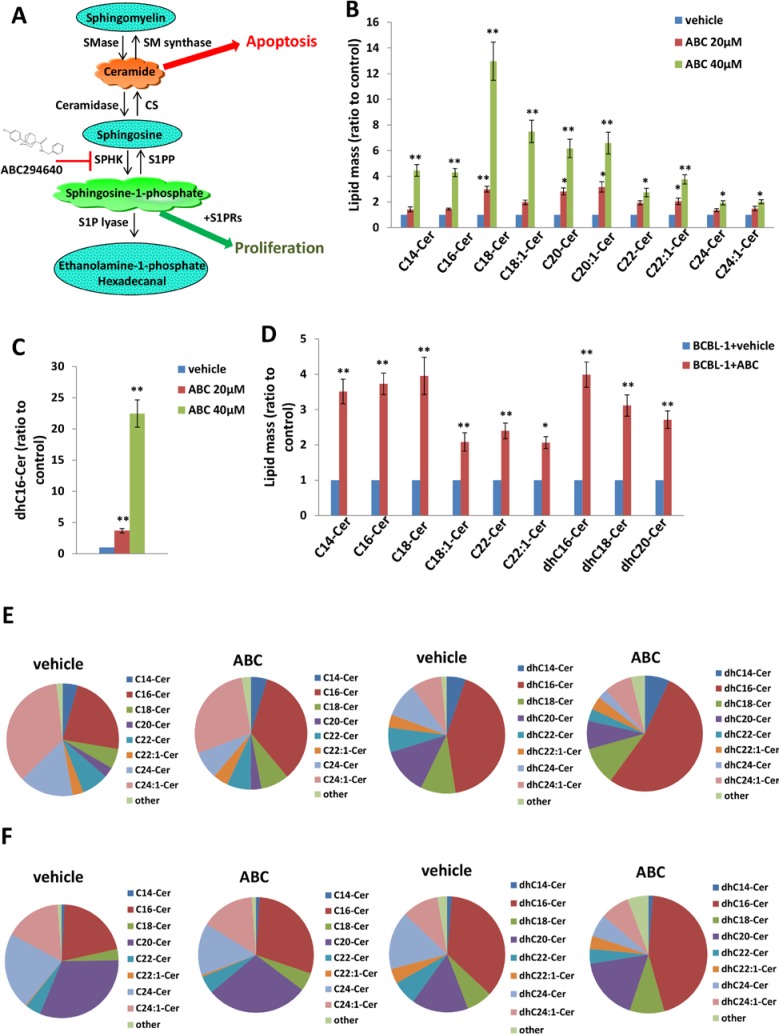
Accumulation of ceramides following targeting of SphK2 within PEL cells **A.** The core pathways of sphingolipid metabolism. **B.–C.** BCBL-1 cells were incubated with the indicated concentrations of ABC294640 (ABC) or vehicle for 16 h, then ceramide and dihydro (dh)-ceramide species were quantified as described in Methods. **D.** NOD/SCID mice were injected i.p. with 10^7^ BCBL-1 cells. Beginning 21 days later, mice were administered 100 mg/kg ABC or vehicle (*n* = 10 per group) i.p. once daily, five days per week, for another 21 days. Live PEL cell lysates were recovered from ascites fractions from each of 3 representative vehicle- or drug-treated mice, and intracellular ceramide and dh-ceramide species quantified as above. Error bars represent the S.E.M. for 2 independent experiments, * = *p* < 0.05; ** = *P* < 0.01. **E.–F.** Relative proportions of specific ceramide and dh-ceramide species within vehicle- or drug-treated PEL cells from *in vitro* **E.** and *in vivo* **F.** experiments are presented.

**Figure 2:**
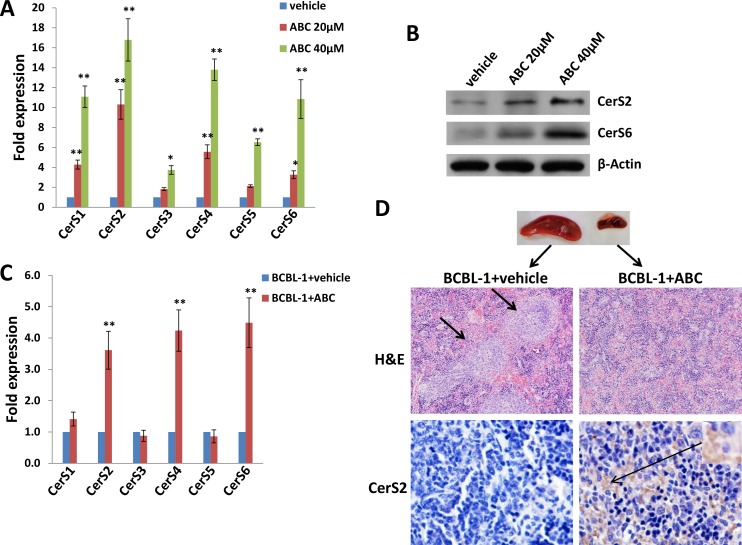
Targeting SphK2 induces upregulation of ceramide synthases within PEL cells **A.–B.** BCBL-1 cells were incubated with the indicated concentrations of ABC or vehicle for 16 h, then transcript **A.** and protein **B.** expression of ceramide synthases (CerS1-CerS6) quantified using qRT-PCR and immunoblots, respectively. **C.** CerS transcripts were quantified using RNA from PEL cells recovered from ascites fractions from each of 3 representative vehicle- or drug-treated mice. Error bars represent the S.E.M. for 2 independent experiments, * = *p* < 0.05; ** = *P* < 0.01. **D.** Spleens from representative vehicle- or drug-treated mice were prepared for routine hematoxylin and eosin (H&E) staining as described in Methods for identification of infiltrating PEL tumors (short arrows), and immunohistochemistry (IHC) was used for identifying CerS2 expression (upper panels, 200x; lower panels, 400x).

We previously reported that a selective inhibitor of SphK2, ABC294640, increased cumulative ceramide levels within KSHV-infected PEL cells [19]. An abbreviated schematic of sphingolipid metabolism is provided (Figure 1A), depicting pathways and potential alterations in sphingolipid metabolism with SphK2 inhibition. More detailed lipidomics analysis performed using a KSHV^+^ body cavity-based lymphoma (BCBL-1) cell line revealed dose-dependent accumulation of multiple long-chain ceramide and dh-ceramide species within these cells with exposure to ABC294640 (Figure 1B–1C). In parallel analyses, we found that BCBL-1 cells recovered from ascites of ABC294640-treated NOD/SCID mice also exhibited increased levels of long-chain ceramide and dh-ceramide species relative to BCBL-1 cells recovered from vehicle-treated control mice (Figure 1D). We also calculated the proportion of individual ceramide species within the total lipid mass of ABC294640-treated PEL cells and noted a relative proportional increase in C16-Cer and dhC16-Cer species both *in vitro* and *in vivo* (Figure 1E–1F). In addition, we found that C16-, C24-, C24:1-, dhC16-, dhC20-Cer accumulate predominantly in BCBL-1 cells *in vitro*, while the C16-, C20-, C24-, C24:1-, dhC16-, dhC20-, dhC24-Cer predominate within BCBL-1 cells recovered from mice (Figure 1E–1F). This demonstrates that common and differential ceramide signatures emerge with SphK2 targeting *in vitro* and *in vivo*. Ceramides are synthesized by a family of CerS enzymes, CerS1–CerS6 [21, 22]. We found that *in vitro* targeting of SphK2 in BCBL-1 cells increased transcript expression for all CerS, confirmed using immunoblots for CerS2 and CerS6 expression (Figure 2A–2B). Furthermore, BCBL-1 cells recovered from ABC294640-treated mice exhibited increased expression of CerS2, CerS4 and CerS6 transcripts relative to BCBL-1 cells from vehicle-treated xenograft mice (Figure 2C). In vehicle-treated xenograft mice, we observed significant splenic enlargement, due to tumor infiltration, relative to ABC294640-treated mice (Figure 2D) [19]. Using immunohistochemistry (IHC), we noted robust expression of CerS2 within splenic tissue from ABC294640-treated mice, with negligible expression within splenic tissue from vehicle-treated mice (Figure 2D). These data suggested a role for specific ceramides and CerS2 in PEL cell death associated with SphK2 inhibition *in vivo*.

### Exogenous long-chain ceramides induce expression of ceramide synthases and apoptosis for PEL cells

**Figure 3:**
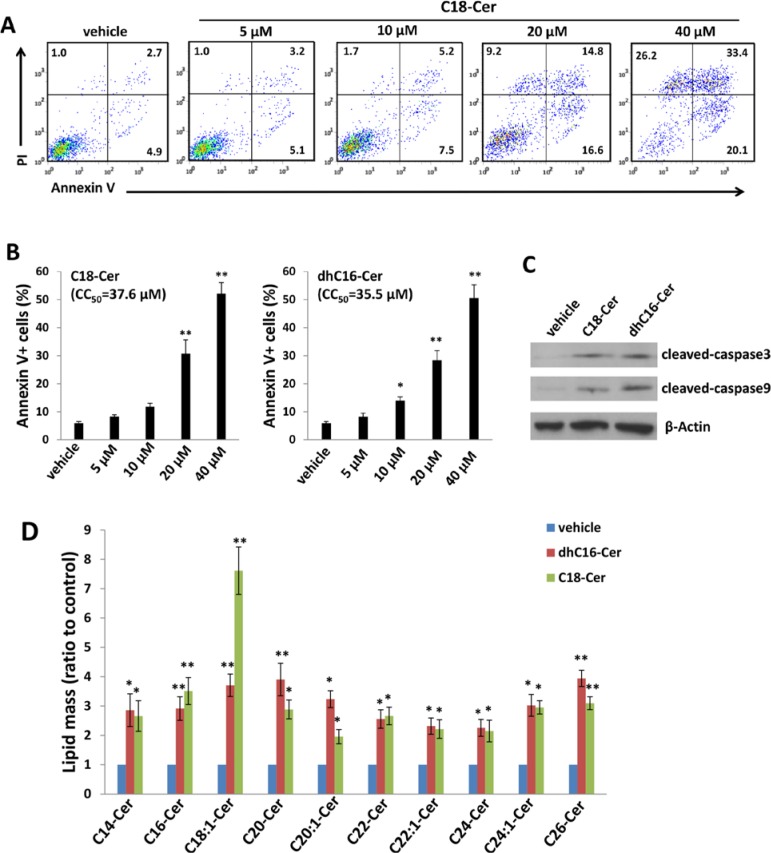
C18-Cer and dhC16-Cer induce accumulation of ceramides and apoptosis for PEL cells **A.–C.** BCBL-1 cells were incubated with the indicated concentrations of C18-Cer, dhC16-Cer or vehicle for 24 h, then apoptosis **A.–B.** and protein expression **C.** quantified as in Methods. **D.** BCBL-1 cells were incubated with C18-Cer (40 μM), dhC16-Cer (40 μM) or vehicle for 24 h, then intracellular ceramide and dh-ceramide species were quantified as described in Methods. Error bars represent the S.E.M. for 3 independent experiments, * = *p* < 0.05; ** = *P* < 0.01.

**Figure 4:**
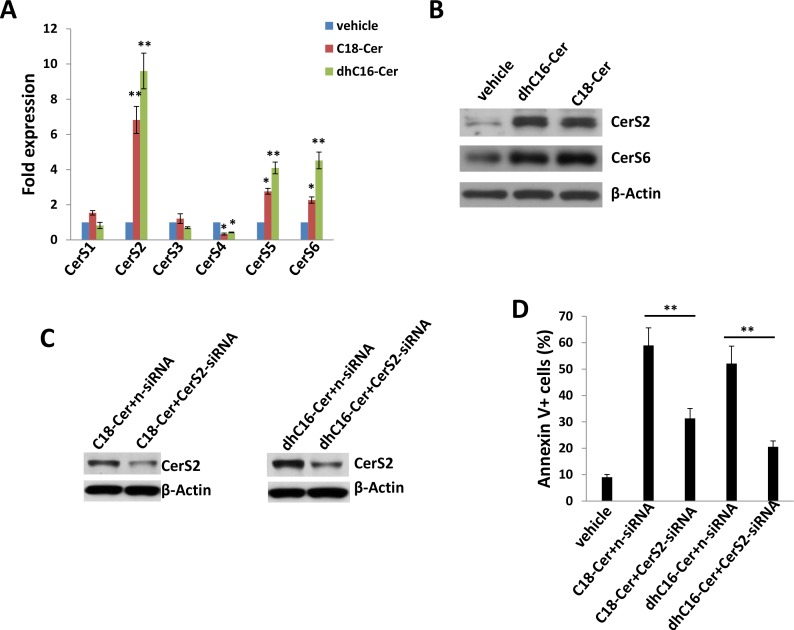
C18-Cer and dhC16-Cer induce expression of ceramide synthases within PEL cells **A.–B.** BCBL-1 cells were incubated with C18-Cer (40 μM), dhC16-Cer (40 μM) or vehicle for 24 h, then transcript **A.** and protein **B.** expression of CerS isoforms quantified using qRT-PCR and immunoblots, respectively. **C.–D.** Cells were transfected with control non-target siRNA (n-siRNA) or *CerS2*-siRNA for 48 h, then incubated with C18-Cer (40 μM), dhC16-Cer (40 μM) or vehicle for 24 h. Protein expression was detected by immunoblots, and cell apoptosis quantified as above. Error bars represent the S.E.M. for 3 independent experiments, * = *p* < 0.05; ** = *P* < 0.01.

Relatively little is known regarding specific roles for individual CerS and their respective ceramide products in cancer development, although available data suggest that CerS6 and its product C16-Cer promote cell survival and tumor growth, while CerS1 and CerS4, and their mutual product C18-Cer, may negatively regulate head and neck cancer growth [3]. Since our initial experiments indicated increased expression of CerS4 and CerS6 within ABC294640-treated PEL cells, we sought to determine whether commercially available ceramides generated by CerS4 (C18-Cer) and CerS6 (dhC16-Cer) directly impact PEL cell survival. Following verification of C18-Cer and dhC16-Cer accumulation within PEL cells with their exposure to these ceramides *in vitro* (Figure S1), we found that C18-Cer and dhC16-Cer induced significant apoptosis for PEL cells in dose-dependent fashion (Figs. 3A–3B and S2), as well as caspase cleavage (Figure 3C), for multiple KSHV^+^ PEL cell lines. Furthermore, exogenous dhC16-Cer induced dose-dependent apoptosis for the following Burkitt's lymphoma cell lines: BL-41 (KSHV^neg^/EBV^neg^), Akata (KSHV^neg^/EBV^+^) and Mutu (KSHV^neg^/EBV^+^) cells (Figure S3). Interestingly, lipidomics analysis indicated that C18-Cer and dhC16-Cer independently increase accumulation of other endogenous long-chain ceramide species within PEL cells (Figure 3D), suggesting that exogenous C18-Cer and dhC16-Cer may regulate expression and/or function of CerS. In fact, we found that C18-Cer and dhC16-Cer independently increased transcript and protein expression for CerS2 and CerS6, as well as transcript expression for CerS5 (Figure 4A–4B). After developing efficient RNA interference for CerS2 in the C18-Cer- or dhC16-Cer-treated PEL cells (Figure 4C), we found that repression of CerS2 partially abrogated the pro-apoptotic impact of both C18-Cer and dhC16-Cer (Figure 4D). Collectively, these data suggest that exogenous long-chain ceramide species induce PEL cell apoptosis, in part through upregulation of specific CerS enzymes.

### Exogenous C18-Cer and dhC16-Cer induce viral lytic gene expression within PEL cells

**Figure 5:**
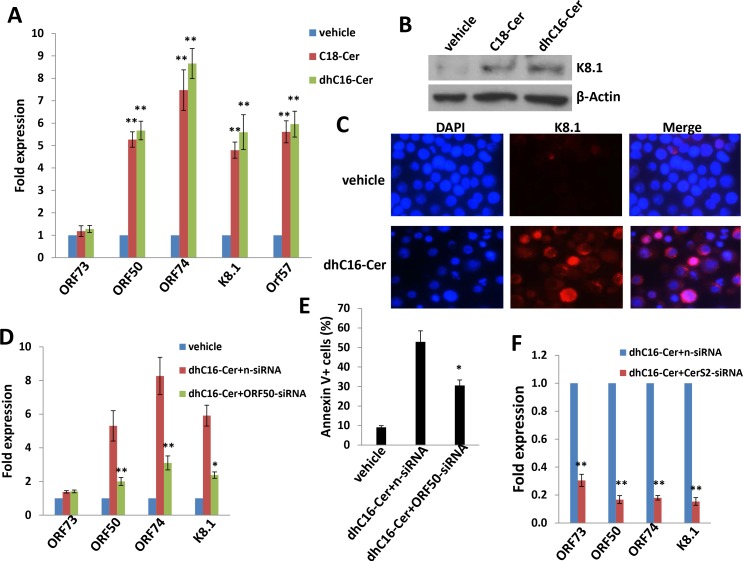
C18-Cer and dhC16-Cer induce KSHV lytic gene expression **A.–C.** BCBL-1 cells were incubated with C18-Cer (40 μM), dhC16-Cer (40 μM) or vehicle for 24 h, then qRT-PCR used to quantify representative KSHV latent (*ORF73)* and lytic transcripts (*ORF50, ORF74, K8.1, ORF57)*. Expression of the viral lytic protein K8.1 was determined using immunoblots and IFA. **D.–E.** BCBL-1 were transfected with control n-siRNA or *ORF50*-siRNA for 48 h, then incubated with dhC16-Cer (40 μM) or vehicle for 24 h. Viral gene expression and cell apoptosis were quantified by qRT-PCR and flow-cytometry, respectively. **F.** BCBL-1 were transfected with control n-siRNA or *CerS2*-siRNA for 48 h, then representative viral transcripts quantified by qRT-PCR. Error bars represent the S.E.M. for 3 independent experiments, * = *p* < 0.05; ** = *P* < 0.01.

To determine whether ceramides induce PEL apoptosis by increasing KSHV lytic reactivation, we quantified representative latent and lytic viral transcripts within BCBL-1 cells in the presence or absence of C18-Cer or dhC16-Cer. We found that either C18-Cer or dhC16-Cer induced expression of viral lytic genes representing all phases of the lytic cycle, while having little impact on expression of KSHV *ORF73* which encodes the latency-associated nuclear antigen (LANA; Figure 5A). These data were supported by observation of increased expression of K8.1, a KSHV envelope protein representing “late” lytic gene expression, when culturing BCBL-1 cells with these exogenous ceramides (Figure 5B–5C). In support of these data, we found that exogenous C18-Cer or dhC16-Cer induced BCBL-1 release of infectious KSHV particles in culture supernatants, as demonstrated by increased KSHV gene expression within KSHV-naïve human umbilical vein endothelial cells (HUVEC) following their exposure to ceramide-treated BCBL-1 supernatants (Figure S4). To determine whether long-chain ceramide-induced viral lytic gene expression is responsible for PEL apoptosis, we performed RNA interference targeting KSHV *ORF50* which encodes the replication and transcription activator (RTA) responsible for the KSHV latent to lytic switch [23]. We observed suppression of “downstream” KSHV lytic gene activation and partial abrogation of apoptosis with dhC16-Cer treatment with knockdown of *ORF50* (Figure 5D–5E). Interestingly, direct silencing of *CerS2* by RNAi incurred more “global” suppression of both latent and lytic genes within dhC16-Cer-treated PEL cells (Figure 5F).

### Exogenous dhC16-Cer suppresses PEL progression *in vivo*

**Figure 6:**
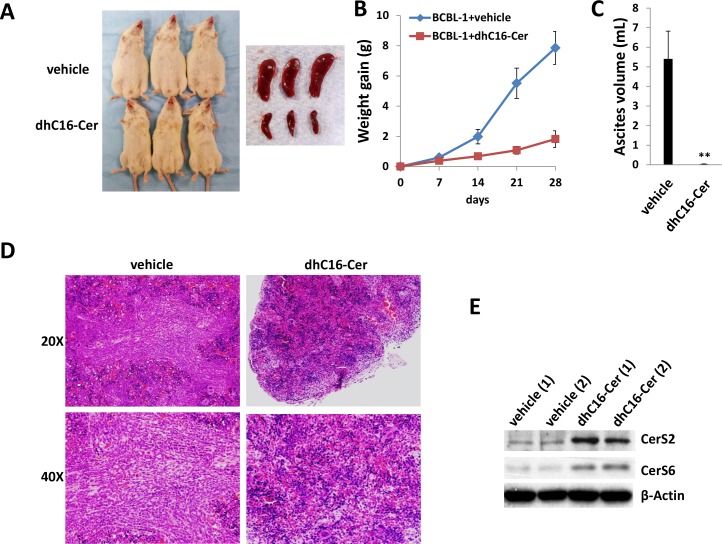
Exogenous dhC16-Cer suppresses PEL tumor progression *in vivo* **A.–C.** NOD/SCID mice were injected i.p. with 10^7^ BCBL-1 cells. Beginning 24 h later, 20 mg/kg dhC16-Cer or vehicle (*n* = 10 per group) were administered i.p. 3x/week, for each of 2 independent experiments. Weights were recorded weekly. Images of representative animals and their respective spleens, as well as ascites fluid volumes, were collected at the conclusion of experiments on day 28. Error bars represent the S.E.M. for 2 independent experiments, ** = *p* < 0.01. **D.** Spleens from representative vehicle- or dhC16-Cer-treated mice were prepared for routine H&E staining for identification of infiltrating PEL tumors. **E.** Immunoblots were used to detect CerS protein expression within splenic lysates from representative 2 vehicle- or dhC16-Cer-treated mice.

Next, we sought to determine whether exogenous long-chain ceramides suppress PEL tumor growth *in vivo* using an established murine xenograft model [34]. We administered dhC16-Cer (or vehicle) intraperitoneally (i.p.) within 24 hours of BCBL-1 cell injection and for one-month duration. We found that dhC16-Cer dramatically suppressed PEL tumor progression over this timeframe (Figure 6A–6C). Using routine IHC, we observed tumor infiltration within spleens of vehicle-treated mice, with only small tumor nests dispersed within spleens of dhC16-Cer-treated mice (Figure 6D). Moreover, although direct ascites tumor analyses were not feasible due to effective suppression of tumor growth in dhC16-Cer-treated animals, immunoblots indicated increased CerS2 and CerS6 expression within splenic lysates from representative animals treated with dhC16-Cer relative to vehicle-treated animals (Figure 6E).

### Exogenous short-chain ceramide species induce PEL apoptosis *in vitro* and *in vivo*

**Figure 7:**
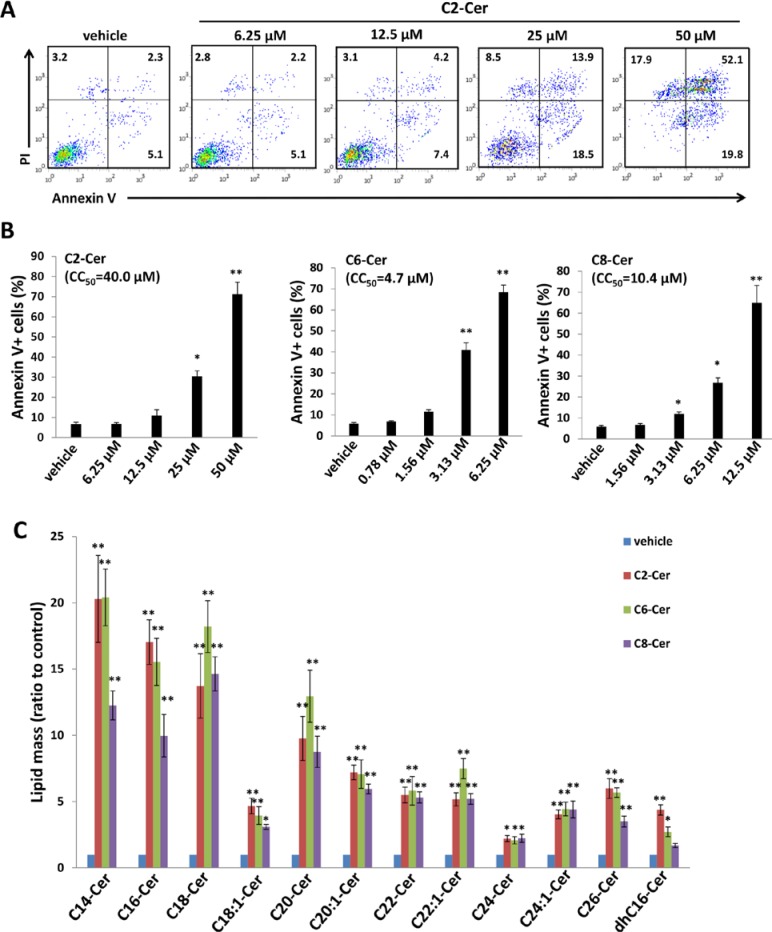
Short-chain ceramides induce ceramide accumulation and apoptosis for PEL cells **A.–B.** BCBL-1 cells were incubated with the indicated concentrations of C2-Cer, C6-Cer, C8-Cer or vehicle for 24 h, then apoptosis quantified as described previously. **C.** Intracellular ceramide and dh-ceramide species were quantified as above. Error bars represent the S.E.M. for 2 independent experiments, * = *p* < 0.05; ** = *P* < 0.01.

In general, short-chain ceramide species cannot be reliably quantified using lipidomics analysis because they are rapidly converted to long-chain ceramides [24]. However, relative to long-chain ceramides, short-chain ceramides may have improved solubility and cell-permeability for therapeutic application [25]. Therefore, we sought to determine whether exogenous short-chain ceramides also induce PEL apoptosis. We found that exogenous C2-, C6- or C8-Cer independently induced dose-dependent apoptosis for KSHV^+^ PEL cell lines, and both C6- and C8-Cer displayed lower inhibitory concentrations (CC_50_) relative to long-chain ceramides such as C18-Cer or dhC16-Cer (Figures 7A–7B and S5). In contrast to long-chain ceramides, we found that Akata and Mutu cells were resistant to C6-Cer-induced apoptosis, while BL-41 cells retained sensitivity to C6-Cer (Figure S6). Similar to their long-chain counterparts, short-chain ceramides also increased accumulation of endogenous long-chain ceramides within BCBL-1 cells (Figure 7C). Of note, the accumulation of intracellular C6- and C8-Cer could be quantified at specific time points following their exogenous administration in cell culture, while C2-Cer could not (Figure S7), suggesting exogenous C2-Cer may be more quickly converted to long-chain ceramides following cell entry.

**Figure 8:**
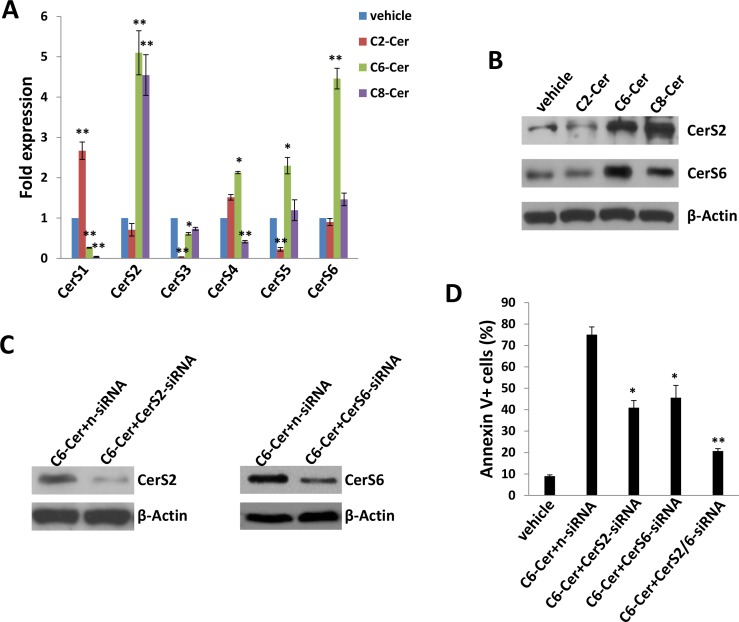
Short-chain ceramides induce expression of ceramide synthases within PEL cells **A.–B.** BCBL-1 cells were incubated with C2-Cer (50 μM), C6-Cer (6.25 μM), C8-Cer (12.5 μM) or vehicle for 24 h, then transcript **A.** and protein **B.** expression of CerS isoforms quantified using qRT-PCR and immunoblots, respectively. **C.–D.** Cells were transfected with control n-siRNA, *CerS2*-siRNA or *CerS6*-siRNA for 48 h, then incubated with C6-Cer (6.25 μM) or vehicle for 24 h. CerS protein expression and cell apoptosis were determined as above. Error bars represent the S.E.M. for 3 independent experiments, * = *p* < 0.05; ** = *P* < 0.01.

Since we found that exogenous long-chain ceramides induce expression of specific CerS in PEL cells, we explored the same principle with exogenous short-chain ceramides. Unlike the selective but uniform impact of C18- and dhC16-Cer on the CerS profile (Figure 4A–4B), C2-, C6- and C8-Cer had varying impacts on CerS transcript profiles (Figure 8A), although C6- and C8-Cer uniformly increased transcript and protein expression of CerS2 and CerS6, albeit to varying degrees (Figure 8A–8B). As previously demonstrated for exogenous C18- and dhC16-Cer, we found that CerS2 silencing reduced BCBL-1 apoptosis during C6-Cer treatment (Figure 8C–8D). In addition, CerS6 silencing incurred similar results, with additive and protective effects for concurrent silencing of both CerS2 and CerS6 (Figure 8C–8D). Furthermore, C2-, C6- and C8-Cer independently increased expression of KSHV lytic transcripts within BCBL-1, although the effect was most pronounced for C2-Cer, and *ORF50* silencing partially suppressed apoptosis induced by exogenous C2-Cer (Figure S8).

**Figure 9:**
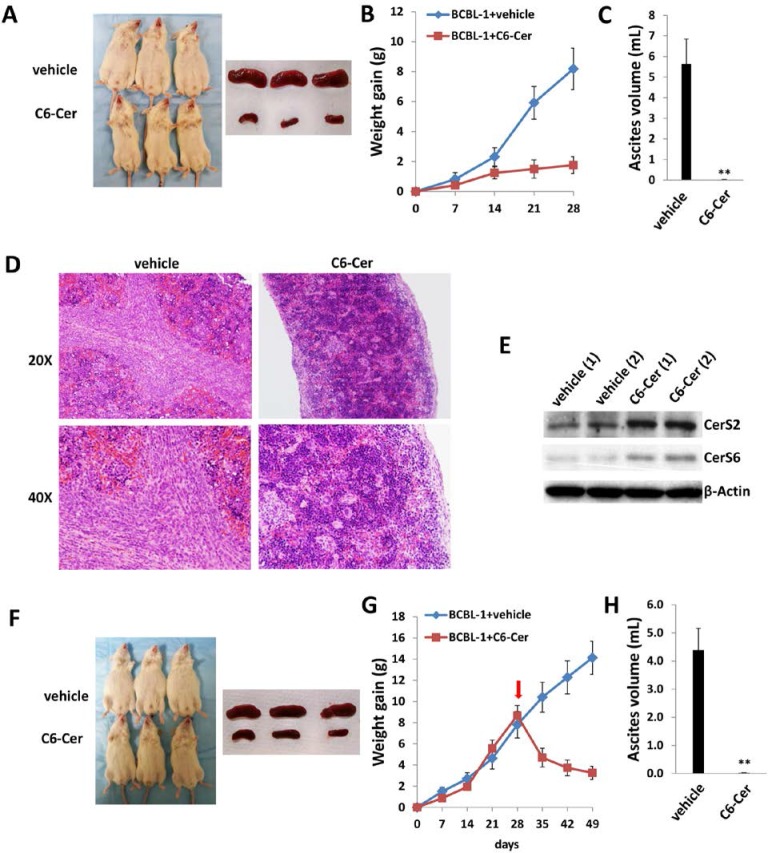
C6-Cer suppresses PEL formation and induces regression of established PEL tumors *in vivo* **A.–C.** NOD/SCID mice were injected i.p. with 10^7^ BCBL-1 cells. Beginning 24 h later, 20 mg/kg C6-Cer or vehicle (*n* = 10 per group) were administered i.p. 3x/week, for each of 2 independent experiments. Weights were recorded weekly. Images of representative animals and their respective spleens, as well as ascites fluid volumes, were collected at the conclusion of experiments on day 28. Error bars represent the S.E.M. for 2 independent experiments, ** = *p* < 0.01. **D.** Spleens from representative vehicle- or C6-Cer-treated mice were prepared for routine H&E staining. **E.** Immunoblots were used to detect CerS protein expression within splenic lysates from representative vehicle- or C6-Cer-treated mice. **F.–H.** NOD/SCID mice were injected i.p. with 10^7^ BCBL-1 cells. Beginning 28 days later, 20 mg/kg C6-Cer or vehicle (*n* = 10 per group) were administered i.p. 3x/week, for an additional 21 days for each of 2 independent experiments. Weights were recorded weekly, and images of representative animals and their respective spleens, as well as ascites fluid volumes, were collected at the conclusion of experiments on day 49.

To validate the potential impact of exogenous short-chain ceramides on PEL tumor growth *in vivo*, we explored whether C6-Cer impacted PEL progression using the same xenograft model. C6-Cer was chosen due to its low CC_50_ for PEL cell lines and better *in vivo* stability (Figures 7B, S5 and S7). Similar to previous experiments, C6-Cer (or vehicle) was administered i.p. within 24 hours of BCBL-1 cell injection and for a duration of one month. We found that C6-Cer dramatically suppressed PEL tumor progression *in vivo* in a manner similar to that observed for dhC16-Cer (Figure 9A–9D). Immunoblots using splenic lysates also indicated that C6-Cer treatment increased CerS2 and CerS6 expression in this compartment (Figure 9E). Additional experiments were conducted wherein C6-Cer therapy was initiated following establishment of PEL tumors (beginning 28 days after BCBL-1 cell injection). Using this approach, C6-Cer-treated mice exhibited significant regression of PEL tumor burden relative to vehicle-treated mice (Figure 9F–9G), with virtually no ascites found in these mice after three weeks of treatment (Figure 9H).

## DISCUSSION

In summary, our findings indicate that individual ceramide species, including both short- and long-chain variants, induce apoptosis for PEL cells. In addition, two potential mechanisms are illuminated: induction of CerS expression and accumulation of other pro-apoptotic ceramides; and induction of pro-apoptotic KSHV lytic gene expression. We have also demonstrated that this can be accomplished by two methods which disrupt the ceramide:S1P ratio, namely inhibition of SphK2 or provision of specific exogenous ceramides. In addition to *de novo* generation of ceramides by CerS, ceramides are generated through metabolism of other complex sphingolipids tightly regulated by specialized enzymes [1, 2, 6]. For instance, ceramides are generated by sphingomyelinases (SMases) responsible for sphingomyelin (SM) hydrolysis [26] or cerebrosidase-mediated GlcCer and galactosylceramide (GalCer) breakdown [27]. Future work should be useful in validating methods and potential clinical applicability of manipulating lipid biosynthesis pathways to induce apoptosis for virus-associated tumors.

Consistent with our findings, published data indicate that provision of exogenous short-chain ceramides results in biologic responses similar to those of ceramide agonists in mammalian and yeast cells [25]. Our observation that specific ceramides induce expression of CerS isoforms and accumulation of other ceramide species in PEL cells are also consistent with studies revealing a direct relationship between exogenous and endogenous ceramides. For example, C8-Cer (*N-octanoyl*-sphingosine) liposomes induced a 10-fold increase in total ceramide levels within canine kidney cells [28]. In another example, C6-Cer triggers sustained endogenous ceramide production in a human myeloid leukemia cell line [29]. In a third report, exogenous C6-Cer induced production of endogenous ceramides within a human lung cancer cell line through recycling of the sphingosine backbone of C6-Cer via deacylation/reacylation [24]. Further work with PEL and other virus-infected tumor cells should illuminate mechanisms for exogenous ceramide activation of lipid biosynthesis pathways responsible for death of these cells, and how these pathways integrate with mechanisms for viral latency that either augment or oppose cell death. Our observation that C6-and C8-Cer demonstrate a lesser impact on KSHV lytic activation (at least relative to other species tested in our experiments), yet still readily induce PEL cell death *in vitro* (both) and *in vivo* (C6-Cer), suggest varying and potentially complimentary mechanisms of PEL cell death induced by exogenous ceramides which might be exploited for developing therapeutic strategies. While beyond the scope of this manuscript, the cell type-specific nature of our observations for Burkitt's lymphoma cell lines also indicate that EBV-infected lymphoma cell lines may be alternatively sensitive or resistant to apoptosis induced by specific ceramide species (possibly related to differential impact of ceramides on EBV gene expression), and that specific ceramide species induce apoptosis for lymphoma cells independent of virus-associated mechanisms.

Due to limitations of solubility and cell-permeability for many ceramide species, ceramide analogues or mimetics have been developed as therapeutic agents. These include pyridinium ceramides (Pyr-Cer) which exhibit a more positive charge (pyridinium ring), allowing for preferential accumulation of these analogues in cancer cells [30] which exhibit a more negative charge in subcellular structures (especially mitochondria) [31]. In two examples, L-t-C6-Pyr-Cer and D-e-C16-Pyr-Cer exhibit significant anti-cancer activity *in vitro* and *in vivo* [30, 32]. Our observation that exogenous ceramides induce KSHV lytic gene suggests that ceramides potentially facilitate KSHV dissemination. Therefore, given that antiviral agents like ganciclovir successfully repress KSHV replication and prevent KSHV-associated neoplasms [33], the combination of ceramide analogues or mimetics with antiviral drugs (such as ganciclovir) may represent a rational therapeutic approach. Regardless, use of ceramides to disrupt lipid biosynthesis pathways regulated by oncoviruses represents a potentially novel and targeted therapeutic strategy for virus-associated lymphoma.

## MATERIALS AND METHODS

### Cell culture and reagents

Body cavity-based lymphoma cells (BCBL-1, KSHV^+^/EBV^neg^) were kindly provided by Dr. Dean Kedes (University of Virginia) and maintained in RPMI 1640 medium (Gibco) with supplements as described previously [34]. BC-1 (KSHV^+^/EBV^+^) and BCP-1 (KSHV^+^/EBV^neg^) cells were purchased from ATCC and maintained in complete RPMI 1640 medium (ATCC) supplemented with 20% FBS. All cells were incubated at 37°C in 5% CO_2_. Burkitt's lymphoma cell lines BL-41 (KSHV^neg^/EBV^neg^), Akata and Mutu (both KSHV^neg^/EBV^+^) were kindly provided by Dr. Dean Kedes (University of Virginia) and Dr. Erik Flemington (Tulane University), respectively, and cultured as described elsewhere [35]. Primary human umbilical vein endothelial cells (HUVEC) were cultured as described previously [36]. All experiments were carried out using cells harvested at low (<20) passages. The 3-(4-chlorophenyl)-adamantane-1-carboxylic acid (pyridin-4-ylmethyl) amide (ABC294640) was synthesized as previously described [37]. C18-Cer and dhC16-Cer were purchased from Avanti Polar Lipids. C2-, C6- and C8-Cer were purchased from Cayman Chemical.

### Cell apoptosis assays

Apoptosis was quantified by flow cytometry using the FITC-Annexin V/propidium iodide (PI) Apoptosis Detection Kit I (BD Pharmingen) according to the manufacturer's instructions. Data were collected using a FACS Calibur 4-color flow cytometer (BD Bioscience).

### RNA interference

*CerS2, CerS6* or KSHV *ORF50* ON-TARGET plus SMART pool siRNA, or negative control siRNA (Dharmacon), were delivered using the DharmaFECT transfection reagent according to the manufacturer's instructions.

### Immunoblotting

Cells were lysed in buffer containing 20 mM Tris (pH 7.5), 150 mM NaCl, 1% NP40, 1 mM EDTA, 5 mM NaF and 5 mM Na_3_VO_4_. Total cell lysates (30 μg) were resolved by 10% SDS–PAGE, transferred to nitrocellulose membranes, and incubated with 100-200 μg/mL of the following antibodies: cleaved-Caspase3, cleaved-Caspase9 (Cell Signaling Technologies), CerS2, CerS6 (Santa Cruz), K8.1 (ABI). For loading controls, lysates were also incubated with antibodies detecting β-Actin (Sigma). Immunoreactive bands were developed using an enhanced chemiluminescence reaction (Perkin-Elmer).

### Immunofluorescence

Cells were incubated in 1:1 methanol-acetone at −20°C for fixation and permeabilization, then with a blocking reagent (10% normal goat serum, 3% bovine serum albumin, and 1% glycine) for an additional 30 minutes. Cells were then incubated for 1 h at 25°C with 1:2000 dilution of a mouse anti-K8.1 monoclonal antibody (ABI) followed by 1:200 dilution of a goat anti-mouse secondary antibody conjugated to Texas Red (Invitrogen). For identification of nuclei, cells were subsequently counterstained with 0.5 mg/mL 4′,6-diamidino-2-phenylindole (DAPI; Sigma) in 180 mM Tris-HCl (pH 7.5). Slides were washed once in 180 mM Tris-HCl for 15 minutes and prepared for visualization using a Leica TCPS SP5 AOBS confocal microscope.

### qRT-PCR

Total RNA was isolated using the RNeasy Mini kit (QIAGEN), and cDNA was synthesized from equivalent total RNA using a SuperScript III First-Strand Synthesis SuperMix Kit (Invitrogen) according to the manufacturer's instructions. Primers used for amplification of target genes are displayed in Supplemental Table 1. Amplification was carried out using an iCycler IQ Real-Time PCR Detection System, and cycle threshold (Ct) values were tabulated in duplicate for each gene of interest in each experiment. “No template” (water) controls were used to ensure minimal background contamination. Using mean Ct values tabulated for each gene, and paired Ct values for β*-actin* as a loading control, fold changes for experimental groups relative to assigned controls were calculated using automated iQ5 2.0 software (Bio-rad).

### Quantification of sphingolipids

Quantification of ceramide and (dh)-ceramide species was performed using a Thermo Finnigan TSQ 7000 triple-stage quadruple mass spectrometer operating in Multiple Reaction Monitoring positive ionization mode (Thermo Fisher Scientific). Quantification was based on calibration curves generated by spiking an artificial matrix with known amounts of target standards and an equal amount of the internal standard. The target analyte:internal standard peak area ratios from each sample were compared with the calibration curves using linear regression. Final results were expressed as the ratio of sphingolipid normalized to total phospholipid phosphate level using the Bligh and Dyer lipid extract method [38].

### PEL xenograft model

10^7^ BCBL-1 cell aliquots were diluted in 200 μL sterile PBS, and 6-8 week-old male non-obese diabetic/severe-combined immunodeficiency (NOD/SCID) mice (Jackson Laboratory) received intraperitoneal (i.p.) injections with a single aliquot. For drug delivery, dhC16-Cer or C6-Cer was diluted in sterile PEG:DMSO (1:1) (Sigma) to achieve 100 μL total volume. The drug, or vehicle alone, was administered using an insulin syringe for i.p. injections. Drug was administered initially at either 1 day or 28 days after BCBL-1 injections, 3 times/week. Two experiments, with 10 mice per group for each experiment, were performed. Weights were recorded weekly as a surrogate measure of tumor progression, and ascites fluid volumes were tabulated for individual mice at the completion of each experiment. All protocols were approved by the Louisiana State University Health Science Center Animal Care and Use Committee in accordance with national guidelines.

### Immunohistochemistry

Formalin-fixed, paraffin-embedded tissues were microtome-sectioned to a thickness of 4 uM, placed on electromagnetically charged slides (Fisher Scientific), and stained with hematoxylin & eosin (H&E) for routine histologic analysis. Immunohistochemistry was performed using the Avidin-Biotin-Peroxidase complex system, according to the manufacturer's instructions (Vectastain Elite ABC Peroxidase Kit; Vector Laboratories). In our modified protocol, sections were deparaffinized in xylene and re-hydrated through a descending alcohol gradient. For non-enzymatic antigen retrieval, slides were heated in 0.01 M sodium citrate buffer (pH 6.0) to 95°C under vacuum for 40 min and allowed to cool for 30 min at room temperature, then rinsed with PBS and incubated in MeOH/3% H_2_O_2_ for 20 min to quench endogenous peroxidase. Slides were then washed with PBS and blocked with 5% normal goat serum in 0.1% PBS/BSA for 2 h at room temperature, then incubated overnight with a rat monoclonal anti-CerS2 antibody at 1:100 dilution (Santa Cruz) in 0.1% PBS/BSA. The following day, slides were incubated with a goat anti-rat IgG or goat anti-rabbit IgG secondary antibody at room temperature for 1 h, followed by avidin-biotin peroxidase complexes for 1 h at room temperature. Finally, slides were developed using a diaminobenzidine substrate, counterstained with hematoxylin, dehydrated through an ascending alcohol gradient, cleared in xylene, and coverslipped with Permount. Images were collected at 200x, 400x or 600x magnification using a Olympus BX61 microscope equipped with a high resolution DP72 camera and CellSense image capture software.

### Statistical analysis

Significance for differences between experimental and control groups were determined using the two-tailed Student's t-test (Excel 8.0), and p values <0.05 or 0.01 were considered significant or highly significant, respectively. The Cytotoxicity Concentration 50 (CC_50_) was calculated by using SPSS 20.0.

## SUPPLEMENTARY MATERIAL FIGURES AND TABLE


