# Supplementary material for: Ceramides promote apoptosis for virus-infected lymphoma cells through induction of ceramide synthases and viral lytic gene expression
Source: Oncotarget. 2015 Jul 3;6(27):24246–60. doi: 10.18632/oncotarget.4759 (PMC4695183; doi:10.18632/oncotarget.4759)
Supplement: Supplementary file 1 [file oncotarget-06-24246-s001.pdf]

Ceramides promote apoptosis for virus-infected lymphoma cells through induction of ceramide synthases and viral lytic gene expression

Supplementary Material

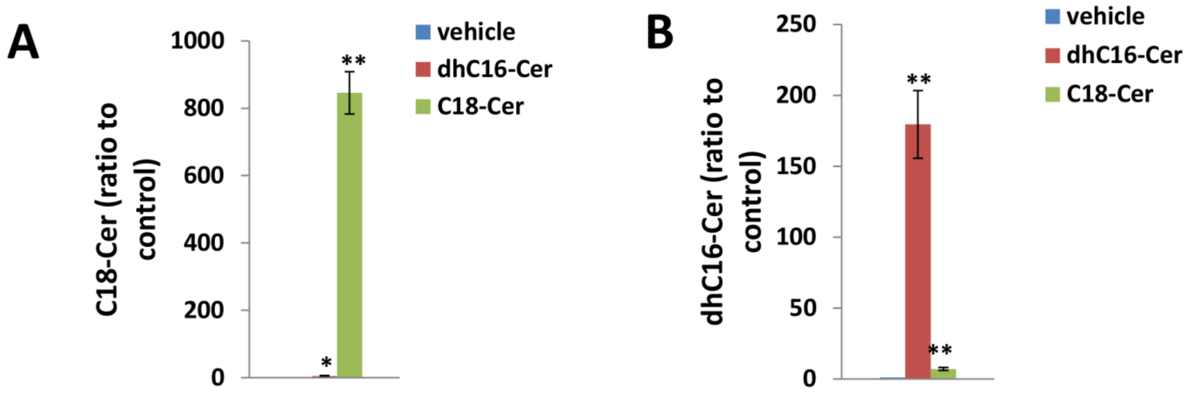

Figure S1: Intracellular accumulation of ceramides within PEL cells *in vitro*. (**A-B**) BCBL-1 cells were incubated with C18-Cer (40 μM), dhC16-Cer (40 μM) or vehicle for 24 h, then intracellular ceramide and dh-ceramide species quantified as described in Methods. Error bars represent the S.E.M. for 2 independent experiments, * = $p<0.05$; ** = $P<0.01$ (*vs* vehicle group).

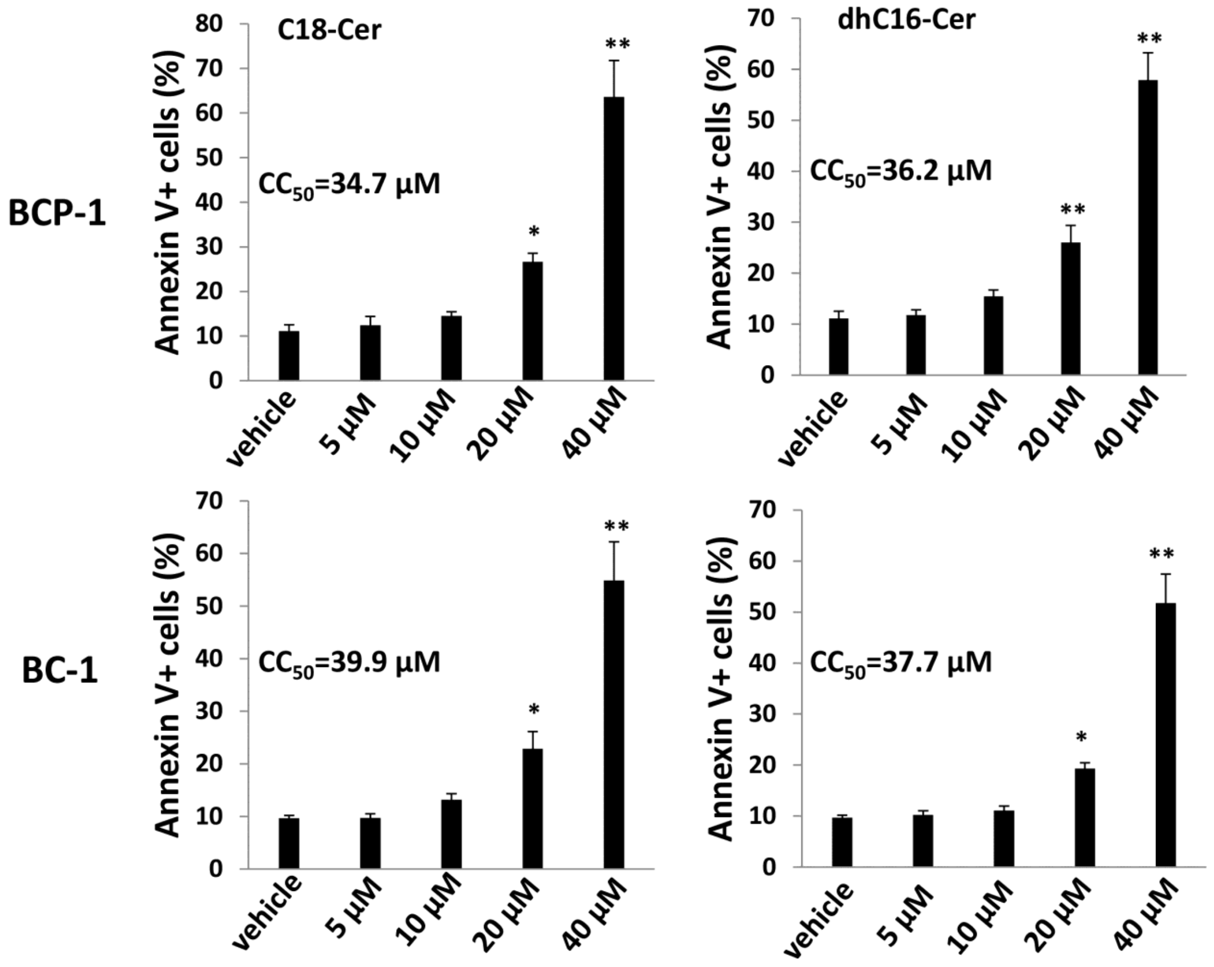

Figure S2: C18-Cer and dhC16-Cer induce apoptosis for KSHV-infected PEL cell lines. KSHV^{+}/EBV^{neg} BCP-1 and KSHV^{+}/EBV^{+} BC-1 cells were incubated with the indicated concentrations of C18-Cer, dhC16-Cer or vehicle for 24 h, then apoptosis was quantified as described in Methods. Error bars represent the S.E.M. for 3 independent experiments, * = $p<0.05$; ** = $P<0.01$.

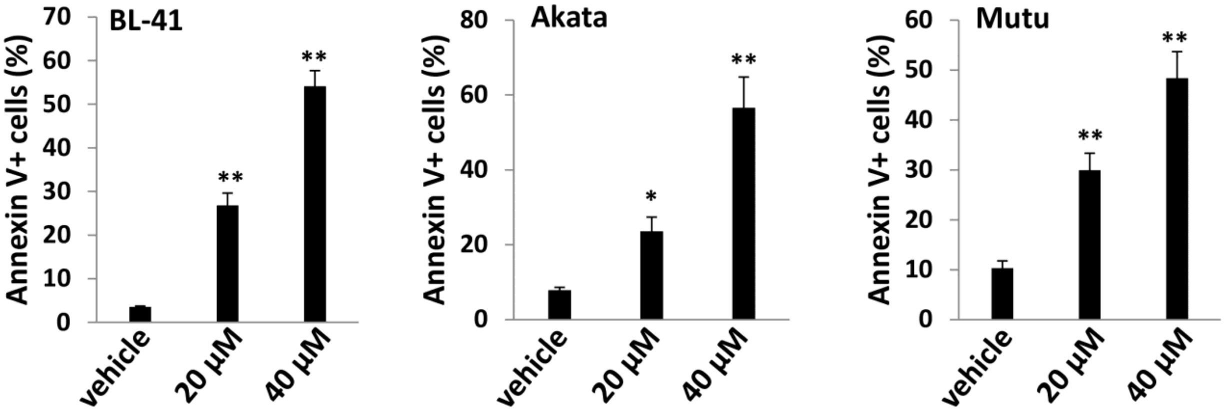

Figure S3: dhC16-Cer induce apoptosis for Burkitt's lymphoma cell lines. Burkitt's lymphoma cell lines BL-41 ($KSHV^{neg}/EBV^{neg}$), Akata and Mutu (both $KSHV^{neg}/EBV^{+}$) were incubated with the indicated concentrations of dhC16-Cer or vehicle for 24 h, then apoptosis was quantified as described in Methods. Error bars represent the S.E.M. for 3 independent experiments, * = $p<0.05$; ** = $P<0.01$.

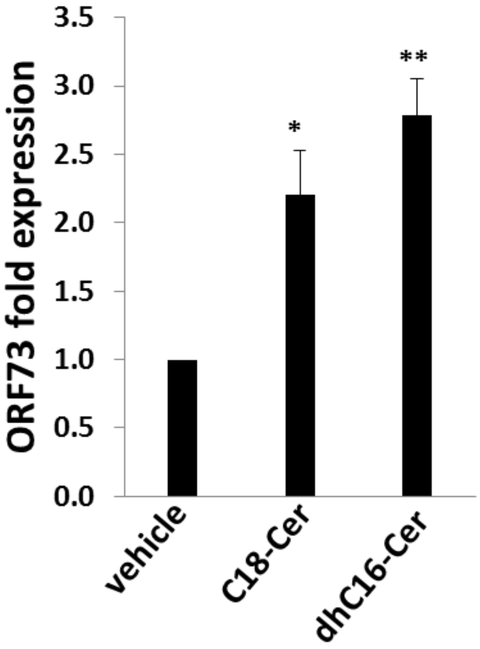

Figure S4: Exogenous C18-Cer and dhC16-Cer induce viral lytic replication within KSHV-infected PEL cells. BCBL-1 were incubated with C18-Cer (40 µM), dhC16-Cer (40 µM) or vehicle for 48 h, then the virion production within culture supernatants quantified as described in Methods followed supernatant incubation with KSHV-naïve HUVEC cells. *ORF73* (LANA) transcripts from each group were quantified by qRT-PCR. Error bars represent the S.E.M. for 3 independent experiments, * = $p<0.05$; ** = $P<0.01$.

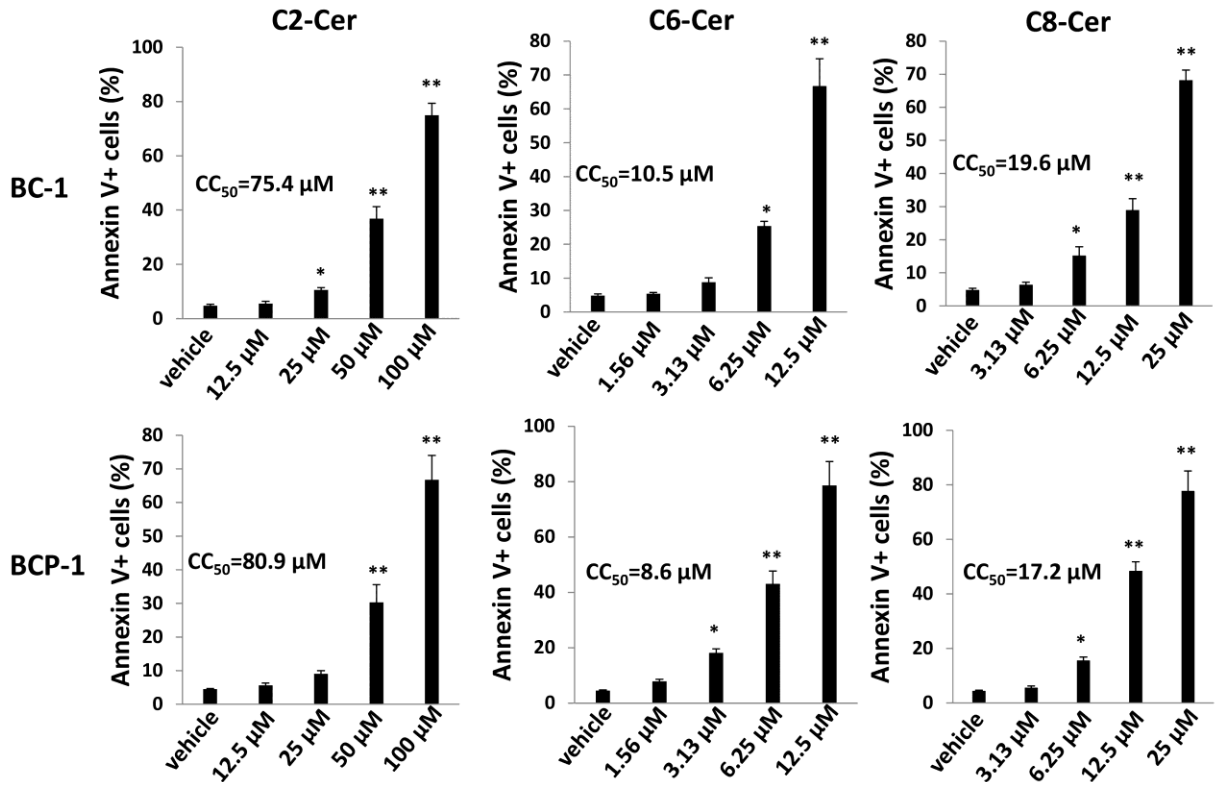

Figure S5: Exogenous short-chain ceramides induce PEL cell apoptosis. KSHV^{+}/EBV^{+} BC-1 and KSHV^{+}/EBV^{neg} BCP-1 cells were incubated with the indicated concentrations of C2-Cer, C6-Cer, C8-Cer or vehicle for 24 h, then apoptosis quantified as described in Methods. Error bars represent the S.E.M. for 3 independent experiments, * = p<0.05; ** = P<0.01.

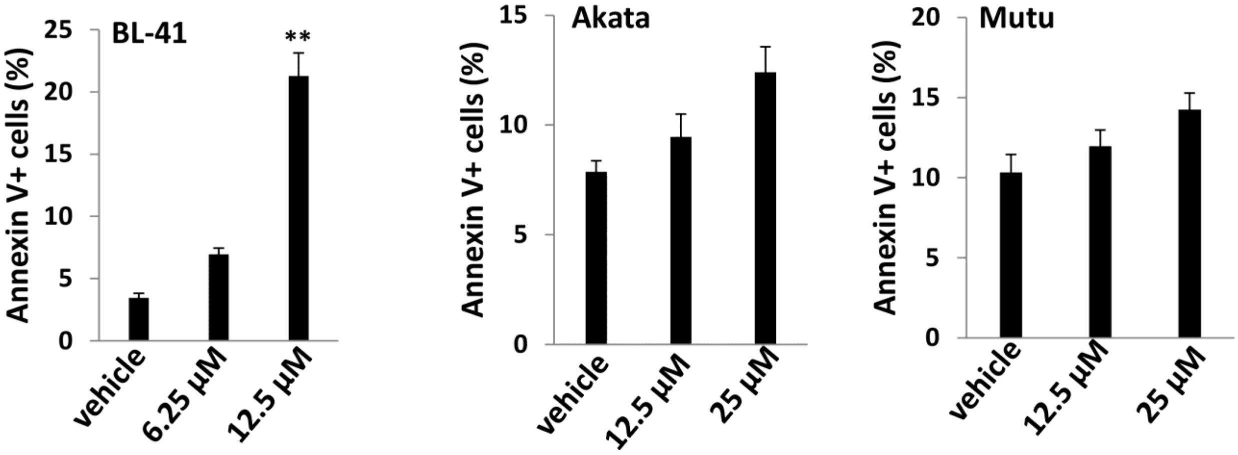

Figure S6: Burkitt's lymphoma cell lines display resistance to exogenous C6-Cer-induced cell apoptosis. KSHV$^+$/EBV$^+$ BC-1 and KSHV$^+$/EBV^{neg} BCP-1 cells were incubated with the indicated concentrations of C6-Cer or vehicle for 24 h, then apoptosis quantified as described in Methods. Error bars represent the S.E.M. for 3 independent experiments, * = $p<0.05$; ** = $P<0.01$.

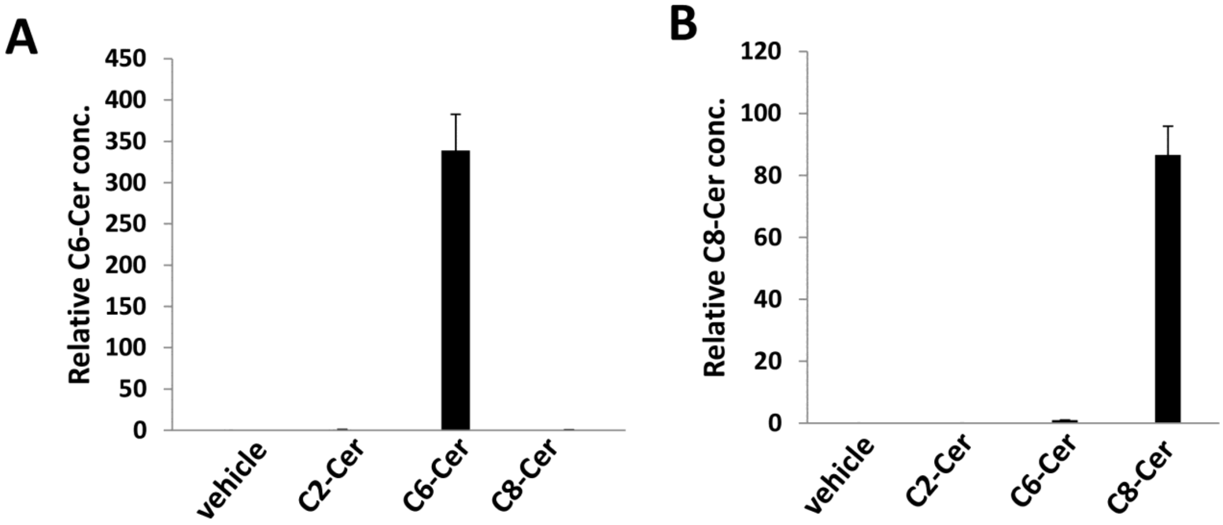

Figure S7: Differential accumulation of short-chain ceramides within PEL cells. BCBL-1 cells were incubated with C2-Cer (50 µM), C6-Cer (6.25 µM), C8-Cer (12.5 µM) or vehicle for 24 h, then short-chain ceremides quantified as described in Methods. Error bars represent the S.E.M. for 2 independent experiments.

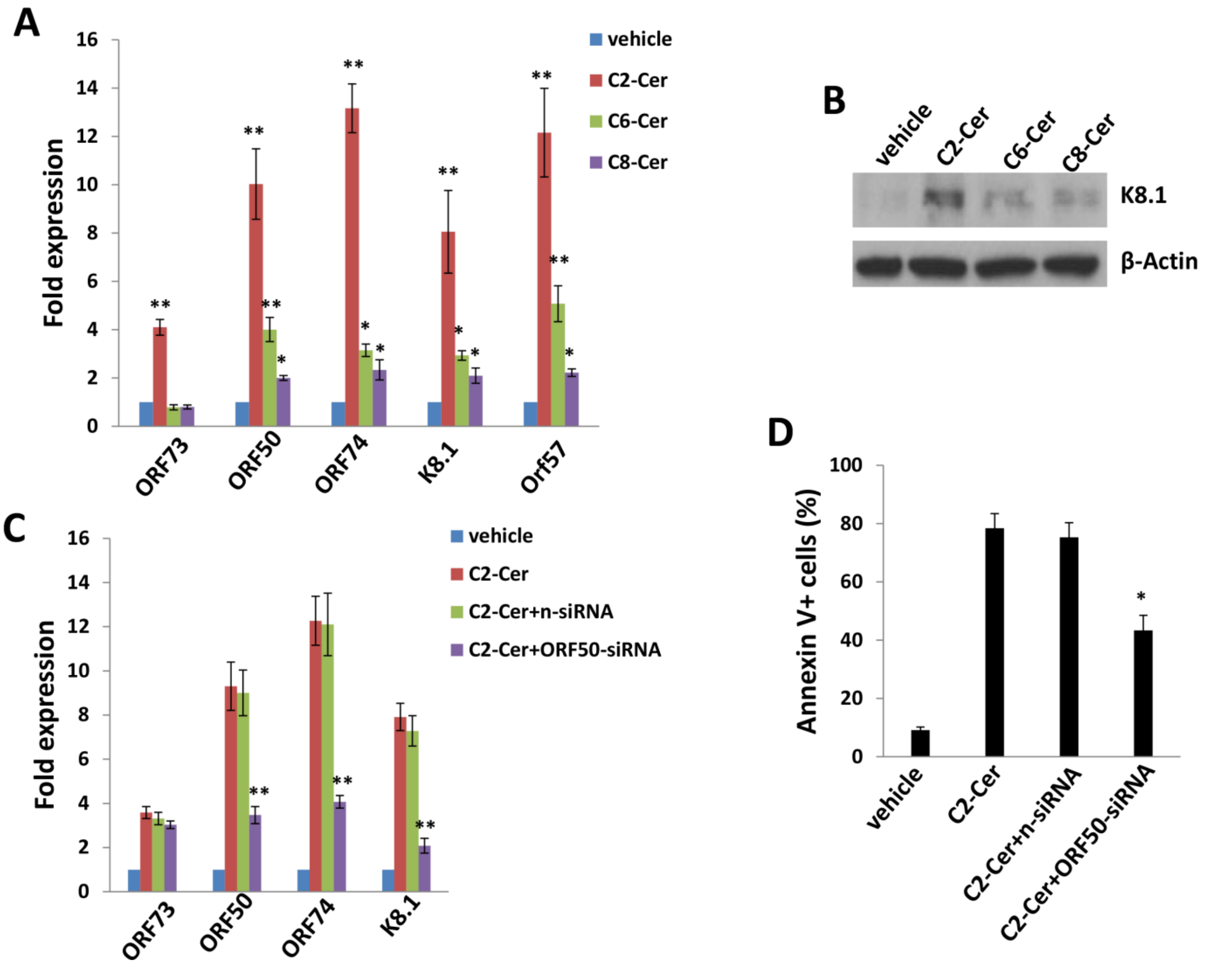

Figure S8: Exogenous short-chain ceramides induce KSHV lytic gene expression. (**A-B**) BCBL-1 cells were incubated with C2-Cer (50 µM), C6-Cer (6.25 µM), C8-Cer (12.5 µM) or vehicle for 24 h, then representative latent (*ORF73)* and lytic (*ORF50, ORF74, K8.1, ORF57)* KSHV transcripts quantified using qRT-PCR. Expression of the viral lytic protein K8.1 was determined using immunoblots. (**C-D**) Cells were transfected with control n-siRNA or *ORF50*-siRNA for 48 h, then incubated with C2-Cer (50 µM) or vehicle for 24 h. Viral gene expression and cell apoptosis were quantified using qRT-PCR and flow-cytometry, respectively. Error bars represent the S.E.M. for 3 independent experiments, * = $p<0.05$; ** = $P<0.01$.

Supplemental Table 1: Primer sequences for qRT-PCR.

**Gene**	**Sequences (5' ⟶ 3')**
Lana	*sense TCCCTCTACACTAAACCCAATA*
	antisense TTGCTAATCTCGTTGTCCC
Rta	*sense TAATGTCAGCGTCCACTCC*
	antisense TTCTGGCACGGTCAAAGC
vGPCR	*sense CATCCGCTGCACTGTTAA*
	antisense GCTTTGTCCTCCTCACCA
K8.1	*sense CACCACAGAACTGACCGATG*
	antisense TGGCACACGGTTACTAGCAC
Orf57	*sense GGGTGGTTTGATGAGAAGGA*
	antisense CGCTACCAAATATGCCACCT
β-actin	*sense GGAAATCGTGCGTGACATT*
	antisense GACTCGTCATACTCCTGCTTG
